# Screening and Characterization of Two Extracellular Polysaccharide-Producing Bacteria from the Biocrust of the Mu Us Desert

**DOI:** 10.3390/molecules26185521

**Published:** 2021-09-11

**Authors:** Zhanfang Xue, Shuting Zhao, Nomin Bold, Jianguo Zhang, Zhimin Hu, Xiaofeng Hu, Ying Gao, Shaolin Chen, Yahong Wei

**Affiliations:** 1 Shaanxi Key Laboratory of Agricultural and Environmental Microbiology, Biomass Energy Center for Arid and Semi-Arid Lands, College of Life Sciences, Northwest A & F University, Yangling, Xianyang 712100, China; xzf@nwafu.edu.cn (Z.X.); zhaoshuting1996@nwafu.edu.cn (S.Z.); nomin@nwafu.edu.cn (N.B.); zhiminhu@nwafu.edu.cn (Z.H.); gaoying666@nwafu.edu.cn (Y.G.); 2 Ministry of Agriculture Key Laboratory of Plant Nutrition and the Agri-Environment in Northwest China, College of Natural Resources and Environment, Northwest A & F University, Yangling, Xianyang 712100, China; zhangjianguo21@nwafu.edu.cn; 3 The Department of Next-Generation Sequencing, Shanghai Personal Biotechnology Co., Ltd., Shanghai 200030, China; huxiaofeng@nwafu.edu.cn

**Keywords:** extracellular polysaccharide, Mu Us Desert, biocrust, bacterial polyphase classification

## Abstract

The extracellular polysaccharide (EPS) matrix embedding microbial cells and soil particles plays an important role in the development of biological soil crusts (BSCs), which is widely recognized as beneficial to soil fertility in dryland worldwide. This study examined the EPS-producing bacterial strains YL24-1 and YL24-3 isolated from sandy soil in the Mu Us Desert in Yulin, Shaanxi province, China. The strains YL24-1 and YL24-3 were able to efficiently produce EPS; the levels of EPS were determined to be 257.22 μg/mL and 83.41 μg/mL in cultures grown for 72 h and were identified as *Sinorhizobium meliloti* and *Pedobacter* sp., respectively. When the strain YL24-3 was compared to *Pedobacter yulinensis* YL28-9^T^ using 16S rRNA gene sequencing, the resemblance was 98.6% and the strain was classified as *Pedobacter* sp. using physiological and biochemical analysis. Furthermore, strain YL24-3 was also identified as a subspecies of *Pedobacter yulinensis* YL28-9^T^ on the basis of DNA–DNA hybridization and polar lipid analysis compared with YL28-9^T^. On the basis of the EPS-related genes of relevant strains in the GenBank, several EPS-related genes were cloned and sequenced in the strain YL24-1, including those potentially involved in EPS synthesis, assembly, transport, and secretion. Given the differences of the strains in EPS production, it is possible that the differences in gene sequences result in variations in the enzyme/protein activities for EPS biosynthesis, assembly, transport, and secretion. The results provide preliminary evidence of various contributions of bacterial strains to the formation of EPS matrix in the Mu Us Desert.

## 1. Introduction

Biocrusts are a profitable and functional soil-focused structure that is crucial to the promotion of soil succession, improvement of surface soil moisture, and prevention of soil erosion. Biocrusts may have an effect on soil properties such as nutrient composition, organic matter content, and material circulation in degraded soil [1]. Microbes are essential components of biogeochemical systems, and they also contribute to soil diversity [2]. According to sampling methods used in other studies involving soil microorganisms, the majority of the soil bacterial community is dormant; however, few microorganisms in the soil need soil available substances to convert it into energy for their own growth, even when optimal growth conditions are chosen, and fewer bacterial communities are active under optimal culture conditions [3]. In this study, the physiological, morphological, and biochemical characteristics of bacteria from the biocrusts were investigated.

Polysaccharides are linked to a number of microbial roles, and their further investigation is worthwhile [4]. Bacterial exopolysaccharides are currently used to supplement conventional plant gums [5]. Exopolysaccharides are used in a variety of industries, including dairy, medicine, and others. They may also have potential applications in wastewater treatment [6]. EPSs have been shown in studies to have a wide range of biological functions, including preventing cell dehydration, protecting cells, and lowering external pressure. This substance can absorb nutrients from the living environment and is part of the general microbial technique used by oligotrophic bacteria to live in nutrient-deficient environments [7]. EPSs may also serve as ion exchangers, limiting the diffusion of certain substances through the biofilm and, thus, preventing direct effects of certain antibacterial drugs on cells [8]. It is critical to discover further microorganisms and ferments that contain polysaccharides, as well as to investigate their application importance.

The amount of EPS biosynthesis produced by soil bacteria is influenced by external factors [9], such as the culture medium components, which are primarily concerned with carbon and nitrogen sources during the culture, as well as the culture environment factors such as pH, temperature, aeration, and initial inoculation, all of which may have an effect on the quantity [10]. The composition of EPS may include homopolymers or heteropolymers, with highly different molecular weights (10 to 1000 kDa) [11]. EPSs are long-chain polysaccharides consisting of branches, repeated sugar units, and sugar derivatives that are formed outside the cells of bacteria and microalgae. Many bacteria produce abundant long-chain capsular polysaccharides, which can maintain a strong association and form a capsule structure enveloping the cell and/or take the form of EPSs that are mostly secreted into the immediate environment [12]. Bacteria produce a variety of EPSs via various biosynthetic pathways, all of which are regulated by a cluster of genes. Most of the biosynthesis of EPSs is regulated by the synthase-dependent pathway. Polysaccharides in various species perform a variety of functions in nature [13]. As a structural polysaccharide, cellulose plays a vital role in regulating cell stability in plants and algae [14]. While intracellular storage compounds such as glycogen, starch, and microbial EPSs are restored in the cell, EPSs are the most effective among them for cell adhesion to soils used as natural binders. Furthermore, cells can defend themselves from stress such as high pH, antibiotics, or dehydration by secreting EPS into the environment. In addition to their natural functions, polysaccharides have long been used in a variety of industrial and technological applications, including cosmetics, food, feed, and pharmaceutics [15]. Because of their distinct chemical structures, their efficacy as biopolymers was found to be high in these applications [16]. Many studies have been conducted on the biosynthesis and control of EPSs from *Sinorhizobium meliloti* and *Rhizobium leguminosarum*, with even more on *Rhizobium meliloti*. Some studies have reported locations of functional genes of extracellular polysaccharides I (EPS I) and II (EPS II), which mainly function in the form of gene clusters (exo/exs gene cluster and exp gene cluster) located on plasmid pSymB, as well as their regulatory genes, which are distributed either on chromosomes (such as mucR, eoxS, exoR, exoD, syrM, phoB and expR) or on the plasmid pSymB (such as exsB, exoX, and wggR). Most of the proteins encoded by regulatory genes are repressors, such as exoS, exoR, exoX, and exsB, which have a negative regulatory role on EPS I [17,18], whereas genes mucR and expR impact EPS II, where each synthesis plays a negative regulatory role. According to previous investigation, some mutations on structural genes of bacterial EPSs not only affect their secretion but also form ineffective nodules. Several studies have shown the synthesis of EPS-regulating genes which play an essential role in the growth of *Rhizobia*, the production of EPS, and the mechanism of metabolism.

The soil bacteria play a dominant role in the composition of biological crusts, due to the extracellular polymers and polysaccharides secreted by bacteria during digestion. Here, the bacteria YL24-1 and YL24-3 isolated from Mu Us Desert were identified. According to the results of the 16S rRNA gene sequence alignments, the strain YL24-1 was detected as a species that produces a high yield of exopolysaccharides, whereas the exopolysaccharide yield of strain YL24-3 was relatively lower than that of strain YL24-1. The associated extracellular polysaccharide-producing genes were identified preliminarily following the design of primers to amplify the genes and TA cloning verification.

## 2. Results and Discussion

### 2.1. The Screening of Two Strains and the Characteristics of Strain YL24-3

Two bacteria, designated as strain YL24-1 and YL24-3, were isolated from a sandy soil in the district of Yulin, Shaanxi province, China. After morphological observation and 16S rRNA gene sequence alignment analysis, strain YL24-1 was identified as *Sinorhizobium meliloti* (99.9% sequence similarity). This result showed that strain YL24-1 was not a potential novel bacterial species according to the 97% sequence similarity criterion.

The strain YL24-3 was characterized as negative in Gram staining, aerobic, nonmotile, non-spore-forming, rod-shaped, and pink in color using a polyphasic taxonomic approach. It grew in a pH ranging from 6.9 to 9.0 (optimized at pH 7.0) and at 15–45 °C (optimized at 30  °C). According to the phylogenetic analysis based on the 16S rRNA gene sequence, the lengths of the 16S rRNA gene sequences of the strain YL24-3 and the strain YL28-9 were 1484 bp and 1520 bp, respectively, and strain YL24-3 was found to be affiliated with the genus *Pedobacter*, showing the highest sequence similarity to *Pedobacter yulinensis* YL28-9^T^ (98.6% sequence similarity) [19]. In our previous study, strain YL28-9 was identified as a new species and was regarded as the reference model strain to conduct the further studies. The nucleotide composition (GC content) of genomic DNA, polar lipid analysis, and DNA hybridization were analyzed in order to determine the homology of both strains. The GC contents of the strains YL24-3 and YL28-9 were designated as 48.7% and 50.4%, respectively. The only respiratory quinone detected in YL24-3 was menaquinone-7 (MK-7). The predominant cellular fatty acids were identified as Iso-C_15:0_, summed feature 3 (C_16:1_ω7*c* and/or C_16:1_ω6*c*), and Iso-C_17:0_ 3-OH. The major polar lipid was phosphatidylethanolamine, confirming YL24-3 as one of the subspecies of the genus *Pedobacter*. In the classification study of the genus *Pedobacter*, Steyn et al. [20] suggested four species: *Pedobacter heparinus*, *Pedobacter piscium*, *Pedobacter africanus* and *Pedobacter saltans.* The classification was emended by adding species named *Pedobacter caeni, Pedobacter roseus, Pedobacter aquatilis* sp., and *Pedobacter namyangjuensis* into the genus *Pedobacter* [21,22,23,24]. The characteristics of the genus *Pedobacter* are generally Gram-negative, strictly aerobic, oxidase-positive and catalase-positive, and rod-shaped bacteria with menaquinone-7 (MK-7) as the major or only respiratory quinone [25,26]. In this study, 16S rRNA gene sequence analysis suggested with the results of morphological, biochemical, and chemotaxonomic characteristics that strain YL24-3 belonged to the genus of *Pedobacter*.

#### 2.1.1. The Phylogenetic Relationship of the *Pedobacter* Strain YL24-3

**Figure 1:**
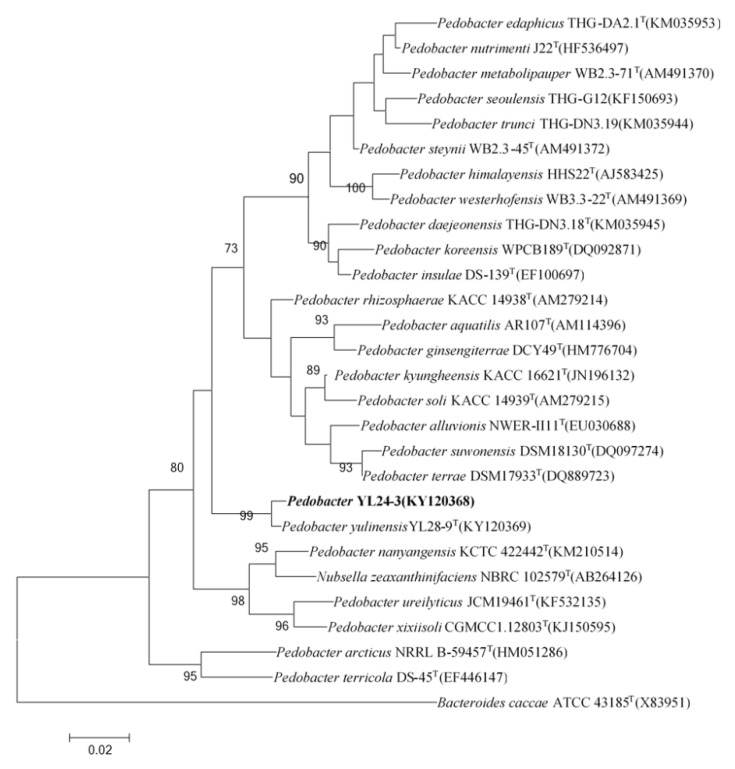
Neighbor-joining tree based on the 16S rRNA gene sequences of strain YL24-3 and related taxa. Bootstrap values are based on 1000 replicates. Only values >70% are shown. Bar, 0.02 substitutions per nucleotide position. The 16S rRNA gene sequence from *Bacteroides caccae* ATCC 43185T was used as the out group.

The phylogenetic relationship between strain YL24-3 and other recognized *Pedobacter* spp. is represented in the neighbor-joining cladogram shown in Figure 1. The nearest relatives of strain YL24-3 were *Pedobacter yulinensis* YL28-9^T^ [19], *Pedobacter* *kyungheensis* KACC 16221^T^ [27], and *Pedobacter soli* KACC 14939^T^ [28]. These types of strains among all other species in the genus *Pedobacter* showed sequence similarities (less than 97%) with respect to strain YL24-3.

#### 2.1.2. Morphological and Physiological Characteristics 

Phenotypic characteristics of the strain YL24-3 are given and compared with those of the four reference strains in Table 1.

**Figure 2:**
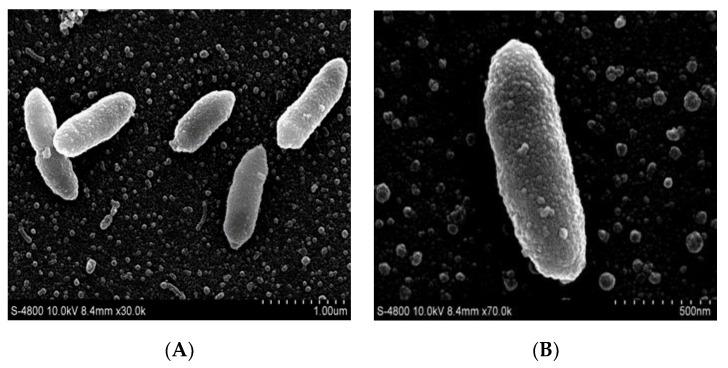
Morphological characterization of strain YL24-3 by scanning electron microscopy (SEM) (scale bar (**A**) = 1.0 µm; (**B**) = 500 nm).

Scanning electron microscopy (SEM) revealed morphological features and confirmed typical bacilliform cells with a rough surface due to irregular granules (see Figure 2; scale bar A = 1.0 µm; B = 500 nm, respectively).

#### 2.1.3. The Physiological and Biochemical Characteristics

Strain YL24-3 could still grow under the normal concentrations of common antibiotics. In addition, both strains held differences in antibiotic resistance, whereby strain YL24-3 was more resistant to Apr and Spe than YL28-9^T^, and strain YL28-9^T^ was more resistant to Str and Kan than YL24-3. The two strains were consistently resistant to Amp, Gen, Ery, Pen, Hyg, and Nyt, which were higher than the normal working concentrations (Table 2).

The tolerance to heavy metal ions in strains YL24-3 and YL28-9^T^ was analyzed as a function of the inhibition zone diameter, which demonstrated varying degrees of tolerance to heavy metal ions as shown in Table 3. With the increase in ion concentration, the tolerance of the same strain to the same metal ion was manifested by the inhibition zone diameter, in which the circle became gradually bigger, indicating tolerance decrease. On the other hand, the strains showed different tolerance to the different metal ions, in which both strains were more resistant to Mn^2+^ and Fe^3+^ but showed poor tolerance to Cd^2+^ and Cr^6+^. Additionally, the tolerance of the strains to Zn^2+^, Pb^2+^, Fe^3+^, and Cd^2+^ was similar, showing a decrease with the increase in ion concentration. Comparing the tolerable difference between these two strains, strain YL24-3 was more tolerant to Cu^2+^ and Mn^2+^ than YL28-9^T^, whereas it was less tolerant to Cr^6+^. This conspicuously illustrated the differences in physiological characteristics between the two strains.

#### 2.1.4. The Results of Polar Lipid and Respiratory Quinone Analyses 

**Figure 3:**
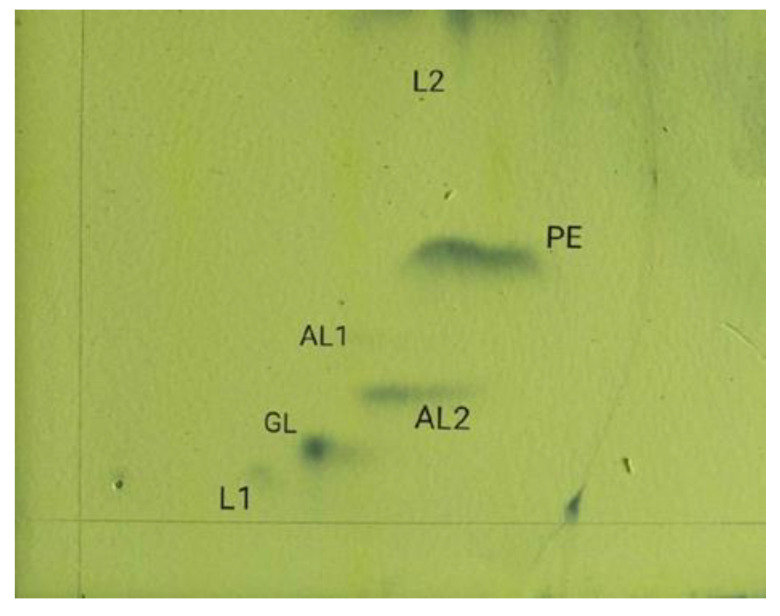
Two-dimensional thin-layer chromatography of polar lipids of strain YL24-3. PE: phosphatidylethanolamine; GL: glycolipid; AL: amino phospholipid; L: unidentified lipids.

The polar lipids of YL24-3 contained unidentified phospholipids, two unidentified lipids, an unknown glycolipid, and two unknown amino phospholipids, which were detected as polar lipids (Figure 3), with phosphatidylethanolamine as the major polar lipid.

The only respiratory quinone identified in the YL24-3 and the reference strains was MK-7. Whole-cell fatty acids were methylated and extracted according to the instructions of MIDI (Sherlock Microbial Identification System, version 6.0) on cells of the YL24-3 and reference strains grown for 48 h at 30 °C. Detailed components and proportions of the YL24-3 and the reference strains are summarized in Table 4, showing that the major fatty acids of the YL24-3 were iso-C_15:0_, summed feature 3 (C_16:1_ ω7c and/orC_16:1_ ω6c), and iso-C_17:0_ 3-OH, which were also found in the reference strains, while the proportions and composition of fatty acids components changed in terms of the different strains.

### 2.2. The Contents of Extracellular Polysaccharides Produced by the Strains

**Figure 4:**
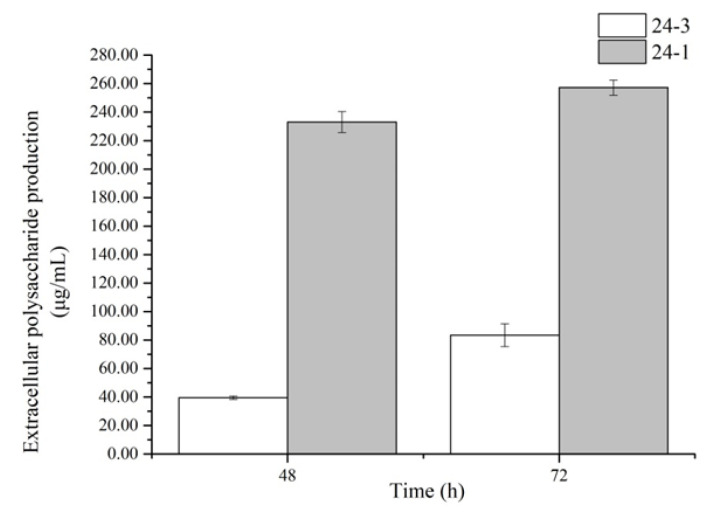
Exopolysaccharide production of strains YL24-3 and YL24-1.

The sugar productions were determined from the selected extracellular polysaccharide-producing bacteria YL24-3 and YL24-1. The contents of the exopolysaccharides were calculated using the glucose standard curve. The results are shown in Figure 4. After 72 h, the exopolysaccharide production became quite low. Too much substance in the late state or during the bacterium splitting period was released by the bacteria, which affected the exopolysaccharide extraction. Therefore, the result of exopolysaccharide production is shown for the first 72 h. The results show that strain YL24-1 had the highest extracellular polysaccharide production at 72 h, with the highest extracellular polysaccharide production reaching 257.22 μg/mL. Under the same fermentation time, the highest exopolysaccharide production of strain YL24-3 was 83.41 μg/mL.

### 2.3. Exopolysaccharide-Related Genes of the Bacteria

Considering the contents of extracellular polysaccharides of the strains above, strains YL24-1 and YL24-3 were selected to analyze their genes related to extracellular polysaccharide production. After analysis, the EPS-producing genes in the genus of *Pedobacter* from the GenBank were screened. The specific primers were designed and synthesized to amplify the target genes of EPSs in strain YL24-3. Unfortunately, no polysaccharide-producing genes were detected in YL24-3. This result suggests that there may be some other functional genes which were not collected in GenBank involved in producing EPSs and regulating the pathway of production. On the other hand, various functional genes were designated in strain YL24-1, *Sinorhizobium meliloti*. So far, the extracellular polysaccharide synthesis protein, transcription activators, and inhibitors for synthesis are known the *Rhizobium meliloti*. Specific primers were used for PCR amplification. 

**Figure 5:**
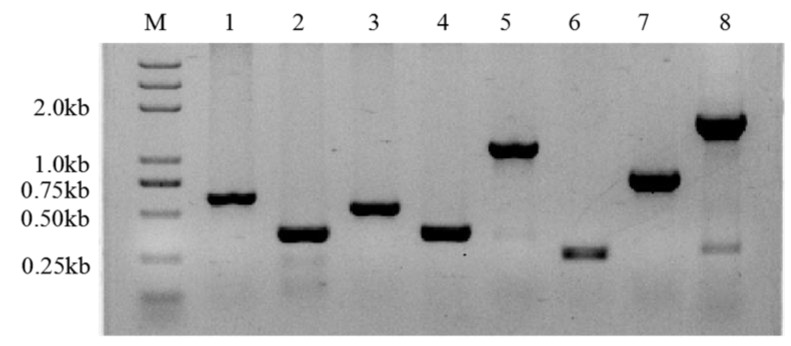
Amplification of extracellular polysaccharide-producing genes. M, marker; 1, gene #152; 2, gene #1371; 3, gene #3901; 4, gene #1802; 5, gene #2164; 6, gene #4312; 7, gene #7310; 8, gene #2936.

According to the specific primers mentioned above, the target fragments were amplified, and the PCR products were detected by gel electrophoresis (Figure 5).

**Figure 6:**
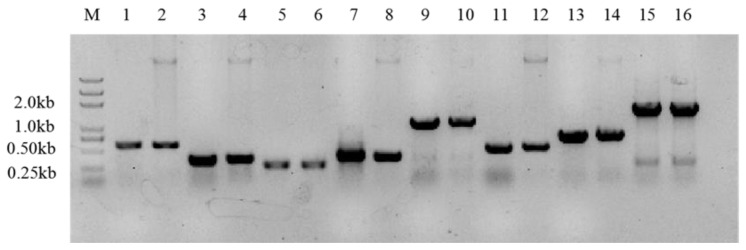
Verification of extracellular polysaccharide-producing genes. M, 2 kb marker; 1, gene #152; 2, Positive control of gene #152; 3, gene #1371; 4, positive control of gene #1371; 5, gene #4312; 6, positive control of gene #4312; 7, gene #1802; 8, positive control of gene #1802; 9, gene #2164; 10, positive control of gene #2164; 11, gene #3901; 12, positive control of gene #3901; 13, gene #7310; 14, positive control of gene #7310; 15, gene #2936; 16, positive control of gene #2936.

After TA cloning and verification (Figure 6), the sequencing results were obtained and compared with the gene sequences of the extracellular polysaccharide-producing gene bank. The genes numbered 1802, 4312, 1371, 2164, 3901, 152, 7310, and 2936 were similar to the genes of *Rhizobium meliloti*. The similarity of the extracellular polysaccharide genes was higher and accounted for 98.93%, 99.01%, 98.90%, 99.00%, 99.80%, 99.16%, 99.74%, and 99.65%, respectively. These genes are certainly responsible for the process of extracellular polysaccharide synthesis. Among these genes, genes 1802 and 1371 are transcriptional regulators (TRs), which affect gene expression at the transcription level. Gene 2164 is a glycosyltransferase (GTFs), which catalyzes the transfer of sugar groups from nucleotide sugar or lipid phosphorus sugar donors to a wide range of acceptor substrates in cells that convert oligosaccharides and polysaccharides. Gene 3901 is an EPS synthesis transcription activator, and gene 4312 is a post-transcriptional regulation inhibitor. These two genes are involved in upstream or downstream regulations of the expression of extracellular polysaccharides at the transcription level. Gene 7310 encodes an extracellular polysaccharide biosynthesis regulatory protein. Gene 152 is an extracellular polysaccharide synthesis protein Exo Y. Moreover, gene 2936 is a cell surface polysaccharide transporter, which is responsible for transporting polysaccharides to designated sites after polysynthesis. These genes play an important role in the processes of polysaccharide synthesis, assembly, transport, and secretion. Gene clusters regulate the production of polysaccharides and the secretion of extracellular polysaccharides at various levels.

## 3. Materials and Methods

### 3.1. Bacterial Strain Screening

Soil bacterial strains YL24-1 and YL24-3 were isolated from sandy soil in the Mu Us Desert in Yulin (34° N, 108° E), Shaanxi province, P.R. China. Using a standard dilution plating technique, serially diluted soil samples were spread on nutrient agar (NA; beef extract, 3 g/L; tryptone, 10 g/L; NaCl, 5 g/L; agar 18 g/L; pH 7.0–7.2) and incubated at 30 °C for 4 days. A single colony of the strain was transferred onto a new plate for purification. The novel strain was routinely cultivated on the fresh NA agar plates. The cultivated YL24-3 was cryopreserved at −80 °C in 50% (*v*/*v*) glycerol. The four reference strains of the *Pedobacter* species were obtained from the Korean Agricultural Culture Collection (KACC) in order to compare their phenotypic and chemotaxonomic characteristics. These strains included *P. ureilyticus* JCM 19461^T^ obtained from the Japan Collection of Micro-organisms (JCM), *P. xixiisoli* CGMCC 1.12803^T^ obtained from the China General Microbiological Culture Collection Center (CGMCC), ‘*P. zeaxanthinifaciens*’ NBRC 102579 obtained from the NITE Biological Resource Center (NBRC), and *P. yulinensis* YL28-9^T^ isolated by our research group.

### 3.2. The Characteristics of Pedobacter Strain YL24-3

#### 3.2.1. Determination of 16S rRNA Gene Sequencing and Phylogenetic Analysis

Genomic DNA extraction of strain YL24-3 was performed using a DNA extraction kit (TIAN-gen). The 16S rRNA gene was amplified by PCR using the universal primers 27F and 1492R [29], carried out as described by Rainey et al. [30] and then double-checked by sequencing both strands. The pairwise sequence alignment similarity was calculated using the EzBiocloud server (https://www.ezbiocloud.net/ (accessed on 19 September 2018)) [31]. The phylogenetic analysis was carried out using MEGA version X with the neighbor-joining (NJ), maximum-likelihood (ML), and maximum-pariony (MP) models after using the multiple sequence alignment program Clustal_W [32]. Statistical support for the branches of the phylogenetic trees were determined using bootstrap analysis based on 1000 replications [33].

#### 3.2.2. Determination of G + C Content 

The genomic DNA of the strain YL24-3 was extracted using the bacterial total DNA extraction kit [34], and the quality of DNA was detected by spectrophotometer to assure the amount of total DNA (up to 20 mg). The DNA was sent to a company called Beijing Novogene Technology to determine the whole genome sequence of the bacteria, and the G + C content was calculated for the obtained sequence.

#### 3.2.3. Morphological and Physiological Characteristics

Cell morphology was examined using a transmission scanning electron microscope (SEM). The growth of strain YL24-3 was tested at 30 °C for 2 days on NA, LB agar, R2A agar, nutrient agar, and tryptic soy agar. The growth of strain YL24-3 was assessed on NA and NB media (NaCl free) at different temperatures (10, 15, 20, 25, 28, 30, 35, 37, 42, and 45 °C). The tolerance to NaCl concentration was determined with 0–7.0% (*w*/*v*) NaCl (with increments of 0.5%) using 20% (*w*/*v*) NaCl to adjust NaCl concentrations. Growth at different pH values (pH 3.0–11.0 at 1.0 pH unit intervals) was evaluated in NB medium for 3 days. NB media below pH 6.0, at pH 6.0–8.0, and at pH 9.0–11.0 were prepared using the following buffer systems: pH 3.0–6.0, 0.1 M H_3_PO_4_/Na_2_HPO_4_; pH 6.0–8.0, 0.1 M KH_2_PO_4_/0.1 M NaOH; pH 9.0–10.0, 0.1 M NaHCO_3_/0.1 M Na_2_CO_3_; pH 11.0, 0.05 M Na_2_HPO_4_/0.1 M NaOH [35]. After sterilization at 121 °C for 20 min, the pH values were readjusted when necessary. Cell motility was tested by stab culture in semi-solid medium. The activities of catalase and oxidase were evaluated by bubble production in 3% (*v*/*v*) H_2_O_2_ solution and 1% (*w*/*v*) *N*,*N*-dimethyl-*p*-phenylenediaminedihydrochloride solution with a fresh colony grown for 2 days in NA [19]. The bacteria were incubated on DNase agar (tryptone, 15.0 g/L; peptone, 5.0 g/L; NaCl, 5.0 g/L; DNA, 2.0 g/L; CaCl_2_, 0.02 g/L; agar 12 g/L) in order to examine the hydrolysis activity of DNA, as well as for further incubation of the bacterial colonies, which were then covered with 1 mol/L HCl for 1 h on the agar plate. After removing the excess acid with a pipette, a clear area around bacterial colonies showed DNase activity. Hydrolysis of starch (0.2%, *w*/*v*), casein (5%, *w*/*v*), l-tyrosine (0.5%, *w*/*v*), Tween-80/20 (1%, *w*/*v*), and CM-cellulose (0.5%, *w*/*v*) was evaluated according to previously described methods [36]. Cells were cultivated at 30 °C for 48 h using API50CH and API20NE kit (BioMerieux, Paris, France) to determine the characteristics of cell metabolism according to the manufacturer’s instructions.

#### 3.2.4. Analysis of Physiological and Biochemical Characteristics

Ampicillin, Streptomycin, chloramphenicol, gentamycin, erythromycin, spectinomycin, kanamycin, apramycin, penicillin, hygromycin, and nystatin were selected to test the antibiotic resistance of strains YL24-3 and YL28-9^T^. In addition, heavy metal ions, i.e., Cu^2+^: CuSO_4_·5H_2_O; Zn^2+^: ZnSO_4_; Pb^2+^: Pb(NO_3_)_2_; Mn^2+^: MnSO_4_; Fe^3+^: FeCl_3_; Cd^2+^: CdSO_4_, and Cr^6+^: K_2_Cr_2_O_7_, were used to analyze the tolerance of strains YL24-3 and YL28-9^T^. The inhibition zone diameter exhibited varying degrees of tolerance to heavy metal ions [37].

#### 3.2.5. Polar Lipid and Respiratory Quinone Analysis

Polar lipids of YL24-3 were extracted and analyzed as described by Minnikin [38]. Strain YL24-3 was cultured in LB medium at 30 °C to the exponential growth phase. Cells were collected by centrifuge at 12,000 rpm at 4 °C, and the cell mass was dried using a vacuum freeze-drying apparatus [39]. Cell masses of the different strains were harvested from cultures grown on LB for 2–3 days at 30 °C. Polar lipids were separated by two-dimensional TLC with Silica 60 F254 plates (10 × 10 cm). Plates were activated at 110 °C for 30 min and cooled at room temperature. The following solvent systems were used to examine polar lipids: (A) chloroform/methanol/water (65:25:4, by volume); (B) chloroform/methanol/acetic acid/water (80:12:15:4, by volume). The total lipids were detected by spraying with 10% (*w*/*v*) molybdophosphoric acid and heating at 120 °C for 10 min. The amino lipids, glycolipids, and phospholipids were detected by spraying with 0.2% (*w*/*v*) ninhydrin, with heating at 120 °C for 5 min, α-naphthol/sulfuric acid, with heating at 120 °C for 5 min, and molybdenum blue reagent (at room temperature), respectively [40].

The respiratory quinones of YL24-3 and reference strains were extracted with methanol and analyzed by HPLC as described by Collins and Xin [41,42].

### 3.3. Determination of the Contents of Extracellular Polysaccharides Produced by Bacteria YL24-1 and YL24-3

The selected extracellular polysaccharide-producing strains YL24-3 and YL24-1 were taken out of the refrigerator at −80 °C for activation and culture. The solution mentioned above was cultured in 100 mL of liquid medium LB in triplicate [43]. The cultured strains in the shaking conditions mentioned above were sampled after fermentation for 48 h and 72 h; then, the contents of extracellular polysaccharides were determined. The extracellular polysaccharides of the two strains were extracted by ethanol precipitation, and the contents of polysaccharides were weighed on a dry weight basis [44].

The exopolysaccharides were fully dissolved in distilled water and then dried to attain a solid state. Then, the phenol–sulfuric acid method was used to determine the contents of the exopolysaccharides. The absorbance value at 490 nm wavelength was measured using a microplate reader, and then the extracellular polysaccharide content was calculated according to the glucose standard curve.

The phenol–sulfuric acid method was used to prepare a glucose as a standard substance to determine the content of extracellular polysaccharides as described above.

### 3.4. Amplification of the Extracellular Polysaccharide-Producing Genes of the Strains

Strains YL24-1 and YL24-3 stored in glycerol were taken out from −80 °C refrigerators, inoculated in sterile LB liquid medium, and cultivated for 24 h. After the strains grew to the logarithmic phase, they were continuously transferred into new media twice. Then, the bacterial DNA was extracted using the DNA extraction kit. The EPS-producing genes in *Rhizobium* and *Pedobacter* were screened from GenBank, and related different specific primers were designed and synthesized on the basis of the gene databank to amplify the target genes related to EPS in strains YL24-1 and YL24-3 [45].

### 3.5. The Structural Genes of Extracellular Polysaccharide-Producing Bacteria YL24-1

A. The exopolysaccharide-producing related genes of *Rhizobium meliloti* were searched in GenBank, along with some other studies that identified genes regarding exopolysaccharide synthesis [46]. Accordingly, corresponding specific primers were designed for verification, and each cell was detected. The presence of exopolysaccharides genes in strain YL24-1 was analyzed to predict the structural genes without biological information from whole-genome sequencing.

B. A plasmid was constructed using TA cloning, and target genes were sequenced. 

C. The functional genes in *Rhizobium meliloti* for producing extracellular polysaccharides were compared to obtain the possible genes of the bacteria. According to the identified extracellular polysaccharide gene of strain YL24-1, the target gene was verified.

## 4. Conclusions

The strains tested in this study were YL24-1 and YL24-3 isolated from sandy soil in the Mu Us Desert in Yulin. They were able to efficiently produce EPSs at levels of 257.22 μg/mL and 83.41 μg/mL, respectively, in cultures grown for 72 h, and they were identified as *Sinorhizobium meliloti* and *Pedobacter* sp., respectively. The EPS-related genes of these two strains were predicted using the NCBI prokaryotic GenBank rather than high-throughput sequencing. A study of the characteristics of extracellular polysaccharide-producing oligotrophic bacteria can be a reasonable predictor of the survival and preliminary interpretation of ESP gene clusters of such bacteria.

## Figures and Tables

**Table 1:** Physiological characteristics of strain YL24-3 and related strains of species of the genus *Pedobacter*.

Characteristic	1	2	3	4	5
Colony color	Pink	Orange	Pinkish-yellow	Yellow	Yellow
Motile	−	−	+	+	−
Catalase	+	+	−	+	+
Oxidase	+	−	−	+	+
Maximum growth temperature (°C)	37	42	40	37	37
pH range for growth	6.0–9.0	6.0–9.0	5.0–10.0	6.0–9.0	5.0–9.0
Growth in 3% (*w*/*v*) NaCl	−	+	+	−	W
**Hydrolysis of**					
DNA	W	+	−	+	+
Casein	−	−	−	+	−
Starch	−	W	+	W	−
Tween-20	W	+	+	−	−
Tween-80	−	+	+	−	−
Nitrate reduction	−	−	−	−	−
Arginine dihydrolase	−	+	+	−	−
Glucose acidification	−	+	−	−	−
Urease	−	+	+	−	−
**Assimilation of**					
d-Mannose	+	−	+	+	+
Malic acid	−	−	−	+	+
Enzyme activities					
Esterase (C4)	−	−	W	W	W
Esterase lipase (C8)	+	+	W	W	W
Cystine arylamidase	+	−	W	W	W
Trypsin	−	−	+	+	+
α-Chymotrypsin	−	−	W	+	−
α-Galactosidase	+	W	W	−	−
β-Galactosidase	−	+	+	−	−
α-Mannosidase	+	−	−	+	+
α-Fucosidase	−	+	−	−	−
**Acid production from**					
d-Arabinose	+	−	−	−	W
l-Arabinose	W	−	−	W	−
d-Galactose	+	W	W	−	+
d-Glucose	+	+	+	W	+
Fructose	W	−	+	−	W
Methyl α-d-glucoside	+	W	−	−	−
Salicin	W	W	W	+	+
Melibiose	W	W	−	−	W
Starch	+	W	+	W	−
Glycogen	−	−	+	W	−
l-Fucose	W	−	+	−	W
DNA G + C content (mol.%)	47.8	50.4	38.4	38.6	36.1

Strains: 1, YL24-3 (this study); 2, *Pedobacter yulinensis* YL28-9^T^ [19]; 3, *P. ureilyticus* JCM 19461^T^ [19]; 4, ‘*P. zeaxanthinifaciens*’ NBRC 102579 [19]; 5, *P. xixiisoli* CGMCC 1.12803^T^ [19]. All data were from this study except where otherwise indicated. +, positive; W, weakly positive; −, negative.

**Table 2:** The growth status of the tested strains under different concentrations of antibiotics.

The Type and Concentration of Antibiotic		Tested Strain	
		YL24-3	YL28-9^T^
Ampicillin (Amp)	30	+	+
	50	+	+
	100	−	−
Streptomycin (Str)	30	+	+
	50	+	+
	100	−	+
Chloramphenicol (Chl)	20	−	−
	30	−	−
	50	−	−
Gentamycin (Gen)	30	+	+
	50	+	+
	100	−	−
Erythromycin (Ery)	150	++	++
	200	+	+
	300	−	−
Spectinomycin (Spe)	50	+	+
	100	+	+
	150	+	−
Kanamycin (Kan)	30	−	+
	50	−	+
	100	−	−
Apramycin (Apr)	30	+	+
	50	+	+
	100	+	−
Penicillin (Pen)	30	+	+
	50	+	+
	100	−	−
Hygromycin (Hyg)	50	++	++
	100	+	+
	200	−	−
Nystatin (Nyt)	5	++	++
	10	+	+
	30	−	−
Control (H_2_O)	0	++	++

−, no bacteria were grown around the filter paper; +, a small number of bacteria were grown around the filter paper; ++, strain was grown vigorously; control: H_2_O.

**Table 3:** The inhibition zone diameter (mm) of the strains under different concentrations (M) of heavy metals.

Metal Ion	YL24-3					YL28-9^T^				
	0.1	0.2	0.3	0.4	0.5	0.1	0.2	0.3	0.4	0.5
Cu^2+^	19	20	30.6	36	36	25.5	28.5	26	42.6	40.6
Zn^2+^	22.6	29	28	36.6	49	21	22.5	27.5	42	51
Pb^2+^	21.6	26	44	40	40	22	21.5	30	37.6	40.6
Mn^2+^	7.6	13	11.6	11.6	16	8.5	10	19.5	31	30
Fe^3+^	10.3	18	19.6	21	23	10.5	16	20	19	23.6
Cd^2+^	58.6	60	64.6	70	75.6	55.5	57	53.5	69	78.6
Cr^6+^	62	78	103.6	102	122	49.5	61	82.5	83	88

Cu^2+^: CuSO_4_·5H_2_O; Zn^2+^: ZnSO_4_; Pb^2+^: Pb(NO_3_)_2_; Mn^2+^: MnSO_4_; Fe^3+^: FeCl_3_; Cd^2+^: CdSO_4_; Cr^6+^: K_2_Cr_2_O_7_.

**Table 4:** Fatty-acid compositions (%) of the strains and the reference species of the genus *Pedobacter*.

Fatty Acids	1	2	3	4	5
**Straight-Chain Saturated**					
C_14:0_	TR	TR	3.3	TR	TR
C_16:0_	1.4	3.5	ND	TR	TR
C_18:0_	ND	1.6	ND	ND	TR
C_19:0_	TR	1.2	ND	TR	ND
C_15:0_	TR	TR	2	TR	TR
C_15:0_	ND	ND	TR	1.4	1.5
C_16:0_	TR	TR	2.7	1.1	1.5
C_17:0_	TR	TR	TR	1.6	TR
**Branched-Chain**					
Iso-C_14:0_	ND	4.8	3.3	TR	TR
Iso-C_15:0_	42.5	31.7	31.8	26.2	28.9
Iso-C_16:0_	TR	TR	TR	3.6	TR
Iso-C_17:0_	TR	TR	ND	TR	TR
Iso-C_15:0_	3.9	3.6	9.9	3.7	3.5
Iso-C_16:0_	TR	ND	1.9	4.6	2.6
Iso-C_17:0_	15.3	6.7	20	13.7	11.5
Anteiso-C_14:0_	ND	1.16	ND	ND	ND
Anteiso-C_15:0_	2.5	3.3	6.7	1.2	1.1
**Monounsaturated**					
C_15:1_ω6c	TR	TR	TR	1.6	3.2
*Summed Feature 1	1.1	1.1	1.1	TR	TR
*Summed Feature 3	17.8	26.2	18.2	19.9	27.1
*Summed Feature 4	1.1	ND	ND	ND	ND
*Summed Feature 9	9.7	6.5	2	4.2	3.5

Strains: 1, YL24-3 (this study); 2, *Pedobacter yulinensis* YL28-9^T^ [19]; 3, *P. ureilyticus* JCM 19461^T^ [19]; 4, ‘*P. zeaxanthinifaciens*’ NBRC 102579 [19]; 5, *P. xixiisoli* CGMCC1.12803^T^ [19]. Values are percentages of the total fatty acids. Fatty acids that account for less than 1% of the total fatty acids are not shown. TR, trace amount (<1.0%); ND, not detected. *Summed features represent groups of two or more fatty acids that could not be separated by gas chromatography with the MIDI system.

## Data Availability

The data presented in this study are available on request from the corresponding author.

## Sample Availability

Samples of the strains are available from the authors.
